# Emerging neurological symptoms after liver transplantation: A 6‐year follow‐up of an adolescent patient with Wilson’s disease

**DOI:** 10.1111/cns.13798

**Published:** 2022-01-07

**Authors:** Wan‐Qing Xu, Rou‐Min Wang, Yi Dong, Zhi‐Ying Wu

**Affiliations:** ^1^ Department of Neurology and Research Center of Neurology in Second Affiliated Hospital, and Key Laboratory of Medical Neurobiology of Zhejiang Province Zhejiang University School of Medicine Hangzhou China

**Keywords:** liver transplantation, neurological symptoms, Wilson's disease

Dear Editor,

Liver transplantation (LT) is a promising treatment modality for patients with Wilson's disease (WD).^1^ According to AASLD, the indication of LT for WD patients is acute liver failure or decompensated cirrhosis unresponsive to chelation treatment.^2^ It is generally believed that LT can benefit patients with hepatic WD and also improve patients with severe neurological symptoms or mixed type WD, but the survival rate of the latter is lower than that of patients with simple hepatic WD.^3^, ^4^ And the efficacy and prognosis of LT in patients with neurological WD are always open to debate,^5^, ^6^, ^7^ and we found that a patient with hepatic WD developed neurological symptoms less than a year after successfully receiving LT for acute liver failure at a young age, and that the patient's neurological symptoms continued to worsen over the next 6 years of regular follow‐up and treatment. The findings suggest that we need to further explore the possible mechanisms underlying the neurological symptoms seen in WD after LT and propose improvements to the subsequent treatment regimen.

An 18‐year‐old female patient was clinically diagnosed with WD at the age of 12 years according to the Leipzig score and was later confirmed by next‐generation sequencing to carry pathogenic variants (p.R778L from father and p.R992L from mother) of two different alleles of *ATP7B*. After obtaining informed consent from her legal guardian, clinical and genetic records were evaluated and analyzed during a six‐year follow‐up. Figure 1 demonstrates the patient's treatment history and change in disease progression from 2015 to 2021.

**FIGURE 1:**
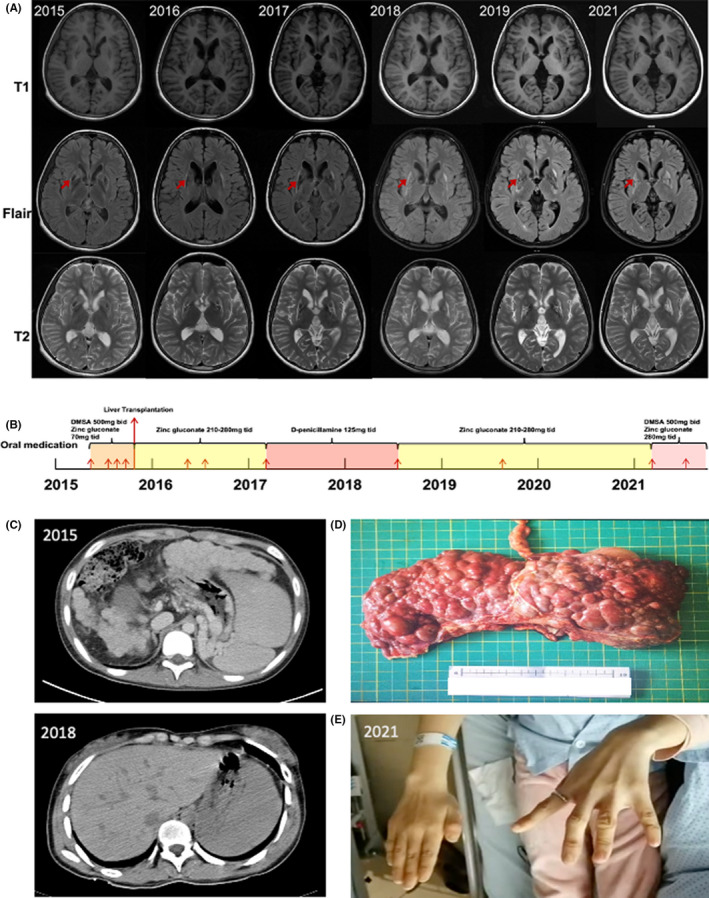
(A) Cranial MRI of the patient from 2015 to 2021, with metal deposition visible in the region of the lenticular nucleus (shown by red arrows). (B) Timeline of the patient's treatment process. Small red arrows stand for single intravenous dose of sodium dimercapto sulfonate (DMPS) copper chelation therapy that the patient received at each hospitalization. DMSA stands for dimercapto succinic acid. (C) CT scan images of the patient's liver in 2015 before LT and in 2018. (D) Intraoperative patient's liver pathology. (E) Patient's left‐hand dystonia manifested in 2021.

The patient was first seen in 2015 for intermittent fatigue, anorexia, and lower extremity edema for 3 years without any neurological symptoms at that time. After being diagnosed with WD and starting copper chelation therapy for 5 months, she underwent total hepatectomy and orthotopic LT for acute liver failure in September 2015. The patient's 24‐hour urine copper and serum ceruloplasmin recovered transiently afterward (Table 1). Based on this, the family considered her disease completely cured and did not follow medical advice to maintain a low‐copper diet. Then the patient revisited 10 months after surgery for newly developed neurological symptoms, including increased muscle tone in the left limbs, limited movement of the left shoulder, elbow and finger joints, dysphagia, dysarthria, salivation, and gait instability. Subsequently, at annual copper chelation follow‐up, the patient's dietary control and medication compliance remained poor, and her relevant laboratory findings progressed (Table 1). Therefore, she was given oral D‐penicillamine treatment in March 2017, and after her 24‐hour urine copper returned to normal, the regimen was changed back to zinc monotherapy again in July 2018 due to the patient's abnormal liver function and decreased serum ceruloplasmin (Figure 1B). The patient's neurological symptoms did not improve significantly throughout the treatment follow‐up, and the UWDRS score gradually increased.

**TABLE 1:** Copper metabolism indexes and UWDRS scale scores of the patient

Time	Serum CP (mg/L)	Urine copper (μg/24 h) before/after i.v. chelation	ALT (U/L)	AST (U/L)	UWDRS N‐score
2015.5	20	1628.8/2577.6	22	41	9
^†^2015.10	251	94.4/–	30	26	–
2016.5	195	–/1025.3	96	84	24
2017.4	191	–/2989.4	71	59	–
2018.7	90	60.6/96.3	180	270	42
2019.8	185	40.5/–	51	58	45
2021.1	163	42.7/–	70	75	49
2021.7	167	37.1/378.5	89	84	58

Note

The normal range of serum CP is 200–600 mg/L; the normal range of 24‐h urine copper is 0–60 μg/24 h; the normal range of ALT is 7–40 U/L; the normal range of AST is 13–35 U/L; the total score of UWDRS is 320, of which the neurological symptom score is 208, with higher score means more severe symptoms.

Abbreviations: ALT, alanine aminotransferase; AST, aspartate aminotransferase; CP, ceruloplasmin; UWDRS, Unified Wilson Disease Rating Scale.

† The patient had liver transplantation in September 2015.

It is generally accepted that copper metabolism indicators should return to normal in WD patients 6 months after LT. In addition, according to the pathophysiological process of copper metabolism in WD, patients should be able to excrete excess copper normally from the liver into the bile and synthesize normal amounts of CP protein after LT.^8^ Therefore, the relatively rare occurrence of progressive worsening neurological symptoms after LT in this patient is difficult to explain. The patient presented mainly with hepatic symptoms and progressed to acute liver failure before LT and did not show neurological symptoms during this period, but after discontinuation of regular medication and dietary control after surgery, progressive neurological symptoms appeared, which were also reflected on the patient's cranial MRI.

We believe that there are two possible reasons for the aggravation of intracranial copper deposition in this case. First, the copper excretion efficacy of the transplanted liver cannot be fully equated with that of the normal human body, as evidenced by the fact that the patient's CP protein, although significantly increased, was still below the lowest value of the normal range. Thus, even with the same diet as in normal subjects, the patient may accumulate more copper. Secondly, not all copper absorbed from the intestine into the blood enters directly into hepatocytes. The non‐ceruloplasmin‐bound copper present in the blood is continuously absorbed by almost all tissues.^8^ In addition to the liver, copper can be deposited in the intracranial, renal, skeletal, and myocardial tissues of patients with WD.^9^ The exact mechanism of how copper enters and is deposited in these tissues is not well understood, but the toxic effects of copper on the tissues cause the corresponding symptoms. In the case of this patient, although the liver was generally functional after transplantation and could excrete most of the copper ions into the bile, excess copper still entered the body through the diet and was deposited in the brain, leading to the progression of neurological symptoms. Therefore, the patient should continue to maintain a low‐copper diet after LT. And in the absence of improvement in neurological symptoms, we also recommend that the patient continue copper chelation therapy.

In our previous study,^10^ 8 patients with WD after LT were followed for 3 to 9 years, whether the patients received LT for reasons of decompensated cirrhosis, acute liver failure, or abnormal liver function with neurological symptoms, and it was found that three patients who maintained long‐term low‐copper dietary control and zinc monotherapy had complete resolution of symptoms, while those who did not have zinc monotherapy and diet control developed other symptoms, including worsening neurological symptoms. Because of the broad and heterogeneous symptom spectrum of WD, treatment is individualized, but based on these studies we believe that patients still need to continue low‐copper dietary control after LT and to take long‐term zinc to reduce the intestinal absorption of copper.

In summary, the therapeutic effect of LT on WD has been widely recognized, but WD is the result of mutations in the *ATP7B* gene in somatic cells throughout the body, and the process of copper deposition and excretion in tissues other than the liver is not fully understood. For the diversity of neurological alterations in WD, we recommend that patients should continue at least low‐copper dietary control and zinc monotherapy after LT to improve long‐term prognosis.

## 1 POTENTIAL CONFLICT OF INTEREST

Nothing to report.
